# Molecular Modeling of the Multiple-Substrate Activity of the Human Recombinant Intra-Melanosomal Domain of Tyrosinase and Its OCA1B-Related Mutant Variant P406L

**DOI:** 10.3390/ijms25063373

**Published:** 2024-03-16

**Authors:** Monika B. Dolinska, Yuri V. Sergeev

**Affiliations:** National Eye Institute, National Institutes of Health, Bethesda, MD 20892, USA

**Keywords:** tyrosinase, molecular docking, molecular dynamic simulation, melanin pathway, OCA1B albinism, mutations

## Abstract

Tyrosinase serves as the key enzyme in melanin biosynthesis, catalyzing the initial steps of the pathway, the hydroxylation of the amino acid L-tyrosine into L-3,4-dihydroxyphenylalanine (L-DOPA), followed by the subsequent oxidation of L-DOPA into dopaquinone (DQ), and it facilitates the conversion of 5,6-dihydroxyindole-2-carboxylic acid (DHICA) into 5,6-indolequinone-2-carboxylic acid (IQCA) and 5,6-dihydroxy indole (DHI) into indolequinone (IQ). Despite its versatile substrate capabilities, the precise mechanism underlying tyrosinase’s multi-substrate activity remains unclear. Previously, we expressed, purified, and characterized the recombinant intra-melanosomal domain of human tyrosinase (rTyr). Here, we demonstrate that rTyr mimics native human tyrosinase’s catalytic activities in vitro and in silico. Molecular docking and molecular dynamics (MD) simulations, based on rTyr’s homology model, reveal variable durability and binding preferences among tyrosinase substrates and products. Analysis of root mean square deviation (RMSD) highlights the significance of conserved residues (E203, K334, F347, and V377), which exhibit flexibility during the ligands’ binding. Additionally, in silico analysis demonstrated that the OCA1B-related P406L mutation in tyrosinase substantially influences substrate binding, as evidenced by the decreased number of stable ligand conformations. This correlation underscores the mutation’s impact on substrate docking, which aligns with the observed reduction in rTyr activity. Our study highlights how rTyr dynamically adjusts its structure to accommodate diverse substrates and suggests a way to modulate rTyr ligand plasticity.

## 1. Introduction

The melanin biosynthesis pathway comprises a series of biochemical reactions occurring in specialized cells known as melanocytes. This pathway is responsible for melanin production, a pigment that contributes to the color of skin, hair, and eyes and plays a crucial role in protecting the skin from harmful ultraviolet radiation. Tyrosinase is a pivotal enzyme in melanin biosynthesis, catalyzing the rate-limiting steps of melanogenesis, which involve the conversion of L-tyrosine into L-3,4-dihydroxyphenylalanine (L-DOPA) via hydroxylation and the subsequent oxidation of L-DOPA into dopaquinone (DQ) [1]. These sequential reactions are crucial for the formation of both eumelanin and pheomelanin, the primary pigments responsible for skin, hair, and eye color. Moreover, in the melanin pathway, tyrosinase catalyzes the oxidation of 5,6-dihydroxyindole-2-carboxylic acid (DHICA) into 5,6-indolequinone-2-carboxylic acid (IQCA) and 5,6-dihydroxy indole (DHI) into indolequinone (IQ) [2]. This suggests tyrosinase could perform functions involving multiple substrates in the melanin biosynthesis pathway.

Disruptions in melanin synthesis can lead to oculocutaneous albinism (OCA), a group of genetic disorders characterized by reduced or absent pigmentation of the skin, hair, and eyes, resulting in increased susceptibility to sunburns, skin cancer, and vision problems (Table S1) [3,4]. OCA1 is the type caused by mutations in the *TYR* gene and is further classified into two subtypes: OCA1A, a more severe form resulting from a complete lack of tyrosinase, leading to white hair, light skin, and light blue eyes with severe visual impairments, including nystagmus, strabismus, and photophobia, and OCA1B, a milder form, in which *TYR* gene mutations partially impair tyrosinase activity, allowing for minimal to moderate melanin synthesis. The clinical presentation of OCA1B varies, with affected individuals exhibiting a spectrum of pigmentation levels. We previously showed that the recombinant intra-melanosomal domain of human tyrosinase (rTyr) and its two temperature-sensitive OCA1B-related mutant variants, R422Q and R422W, are soluble monomeric glycoproteins that exhibit enzymatic activity similar to their in vivo counterparts [5]. Another clinically significant mutation causing OCA1B is the P406L [6,7]. Our in vitro analysis revealed that this missense mutation, which replaces proline with leucine at position 406, reduces catalytic activity compared with the wild-type enzyme because of an alteration in protein conformation and stability [8]. The structural changes caused by the P406L mutation hinder the enzyme’s ability to catalyze melanin synthesis effectively, resulting in skin, hair, and eye hypopigmentation.

Understanding melanin biosynthesis is crucial for human health and disease. Studying how small-molecule ligands interact with rTyr and its P406L mutant variant can reveal genotype-phenotype correlations in OCA1B. In addition to in vivo and in vitro studies, computational methods like molecular docking and molecular dynamics (MD) simulations can provide valuable insights into the biomolecular interactions and mechanisms involved in melanin biosynthesis. For years, molecular docking has been an important tool for drug discovery facilitating the creation of new therapeutics, but it has also been employed to clarify fundamental biochemical processes [9,10]. This in silico approach has been extensively utilized in the field of tyrosinase research, particularly in pharmacological studies focusing on tyrosinase inhibitors that serve as skin-whitening or anti-aging agents [11,12,13,14]. Using molecular docking, Kumari et al. explored glabridin derivatives’ anti-tyrosinase efficacy and effects on melanoma cells [15]. Computational methods have also been used to investigate the effect of mutations on enzyme–ligand interactions; Kamaraj and Purohit studied Tyrp1 mutant variants R326H and R356Q, associated with OCA3 albinism, using MD simulations to understand structural consequences [16].

Previously, we utilized rTyr and several OCA1-related mutant variants to analyze tyrosinase binding during its diphenol oxidase reaction [17,18]. Van’t Hoff temperature-dependent analysis suggested that the association of L-DOPA with rTyr is a spontaneous enthalpy-driven reaction and becomes unfavorable at the final step of dopachrome formation. Additionally, the temperature-dependent kinetics of rTyr and two OCA1B mutant variants (R422Q and P406L) were analyzed using Michaelis–Menten and Van’t Hoff analyses. The results revealed, for the first time, that the association of L-DOPA with R422Q and P406L, followed by dopachrome formation, is a complex reaction supported by enthalpic and entropic forces.

Although these studies provide some explanation for the decreased catalytic activity of mutant variants, some important questions about tyrosinase activity are still not answered. For example, the role of the catalytic cavity and di-copper active site in the recognition of multiple substrates such as L-tyrosine, L-DOPA, DHICA, DHI, and the products of tyrosinase reaction. Our objective is to comprehensively understand the impact of disease-related mutations on enzyme activity and to gain as much knowledge as possible about the mechanism of enzymatic reactions involved in melanin production. Nevertheless, in vitro studies with recombinant proteins, while crucial, have certain limitations. Deriving atomic-level mechanisms of tyrosinase–substrate interactions from in vitro studies can be challenging. Additionally, capturing dynamic information about the movement of enzymes and their substrates during the binding process can be extremely challenging in vitro.

In this study, we used biochemistry, molecular docking, and MD simulations to investigate the multi-substrate behavior of rTyr, the related biomolecular interactions, and the mechanisms of small-molecule ligands binding to rTyr and its P406L mutant variant, distinguishing between substrates and products involved in melanin synthesis. We demonstrated that, of the ligands undergoing MD simulations, only tyrosinase substrates showed steady binding to rTyr’s active site, while reaction products, if properly docked at all, were released from it. We also showed that P406L mutation in tyrosinase can affect substrate docking, leading to altered enzyme–substrate interactions and ultimately impacting the enzyme’s catalytic activity. Our research emphasizes the adaptability of rTyr’s structure in accommodating various substrates and underscores the significance of studying rTyr in comprehending human tyrosinase functionality.

## 2. Results

### 2.1. The Multi-Substrate Activity of rTyr and P406L Mutant Variants In Vitro

**Figure 1:**
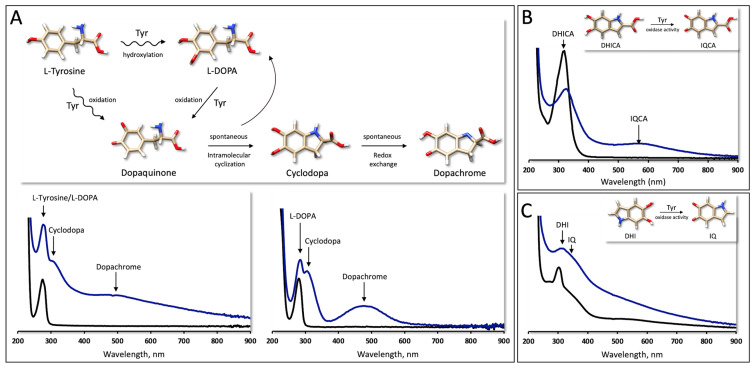
Multi-substrate enzymatic reactions of rTyr. (**A**) Reaction of monophenol and diphenol oxidase activity of Tyr (Top Panel) and enzymatic activity of rTyr (bottom panels). Spectra represent the absorbance profile recorded for wavelengths from 200 to 900 nm at time 0 (black) and after incubation at 37 °C with L-tyrosine (left graph) and L-DOPA (right graph) (blue). Arrows indicate the maximum absorbance of L-tyrosine/L-DOPA (λ_max_~280 nm), cyclodopa (λ_max_ 310 nm), and dopachrome (λ_max_ 475 nm). (**B**) DHICA oxidase activity of rTyr. Spectra represent the absorbance profile recorded at wavelengths from 200 to 900 nm at time 0 (black) and after incubation at 37 °C with DHICA (blue). Arrows indicate the maximum absorbance of DHICA (λ_max_ 325 nm) and IQCA (λ_max_ 560 nm). (**C**) DHI oxidase activity of rTyr. Spectra represent the absorbance profile recorded for wavelengths from 200 to 900 nm at time 0 (black) and after incubation at 37 °C with DHI (blue). Arrows indicate the maximum absorbance of DHI (λ_max_ 305 nm) and IQ (λ_max_ 327 nm). All small molecules were visualized using USCF Chimera. Nitrogen atoms are colored blue, oxygen atoms are colored red, hydrogen atoms are colored white, and carbon atoms are colored tan.

While the melanin pathway involves three enzymes—tyrosinase and two tyrosinase-related proteins, Tyrp1 and Tyrp2 (DCT)—tyrosinase is the only one exclusively essential for melanogenesis. As a key enzyme, it catalyzes the rate-limiting step and the oxidation of L-tyrosine into L-DOPA and DQ, which acts as a subsequent substrate for synthesizing eumelanin (Figure 1A, top panel).

DQ is very reactive and immediately undergoes spontaneous intramolecular cyclization into cyclodopa, which can be oxidized into dopachrome by redox exchange [19]. For this reason, when running, in vitro, the absorbance spectra of an rTyr-catalyzed reaction with L-tyrosine or L-DOPA, instead of DQ, we can only see the λ_max_ values of cyclodopa and dopachrome (310 and 475 nm, respectively) (Figure 1A, bottom panels). From the absorbance spectra of the rTyr-catalyzed oxidation of DHICA, we can observe the emergence of IQCA with λ_max_ at 560 nm (Figure 1B). The DHI oxidase activity of tyrosinase leads to the formation of IQ, which belongs to the highly elusive group of quinones. Given its instability, the maximum absorption presented in the literature varies [20,21,22]. However, using rTyr, a transiently forming IQ with a λ_max_ at 327 nm can be observed (Figure 1C) [23]. The P406L mutant variant reduced the rTyr activity by 72%, 68%, 67%, and 50% for the reactions involving L-tyrosine, L-DOPA, DHICA, and DHI, respectively (Table S2). For both the rTyr and P406L domains, several biochemical reactions are performed with different ligands in the enzyme’s active sites. Our study of multi-substrate activities showed that rTyr mimics, in vitro, the catalytic activities of native human tyrosinase. To gain insight into the molecular mechanism underlying the multi-substrate activity of rTyr, we conducted molecular docking and MD simulations.

### 2.2. In Silico Simulations

#### 2.2.1. rTyr/P406L Docking: Substrates and Products of the Enzymatic Reaction

**Figure 2:**
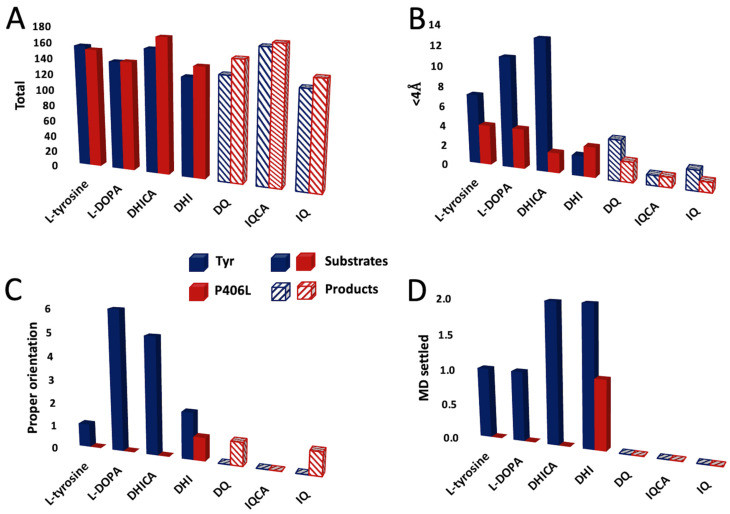
Molecular docking of the substrates and products of the enzymatic reaction catalyzed by the rTyr and P406L mutant variant (**A**) Number of complex formations (total) found after clustering the 25 docking runs of the ligands—substrates (solid bars) and products (stripped bars)—to rTyr (blue) and P406L (red). (**B**) shows the number of ligands bound to the active site in a distance below 4.0 Å from the CuA or CuB atoms. (**C**) shows the number of ligands of which the oxygen of the hydroxyl or carbonyl group is directed toward the active site (proper orientation). (**D**) shows the number of all properly docked structures that remain in optimal alignment when measured throughout 20 ns of the MD simulations.

The interactions of ligands with the key amino acid residues and copper ions present in the active site of the rTyr and P406L mutant variant was examined in silico using molecular docking and MD simulation in YASARA. All docking simulations were performed in both vacuum and water environments to assess how water molecules impact these protein–ligand interactions. When analyzing the potential binding of the substrates (L-tyrosine, L-DOPA, DHICA, and DHI) and products (DQ, IQCA, and IQ) of tyrosinase’s reaction to the rTyr and P406L mutant variant, we derived the same criteria for binding, in that the ligands will be directed with the oxygen from hydroxyl or carbonyl functional groups toward the enzyme active site and have to be located below 4 Å from copper ions A or B. All the interactions were further analyzed by using additional MD simulations to study the dynamic nature of ligand binding and conformational changes in rTyr/P406L–ligand complexes. We adopted the principle that if the ligand remained in a similar position within 20 ns of an MD simulation without exceeding 4 Å, it was considered an optimal alignment and called MD settled (steady complex). In Figure 2, we display the sum of individual docking runs using two forms of ligands (with and without energy minimization) and five timeframes (0, 25, 50, 75, and 100 ns) selected from the MD simulation for both the rTyr and P406L mutant variant models. Given the inability to conduct statistical analysis in this case, we only present a general trend in each panel.

We noted that, when docking in the presence of water, there are more initial complex conformations for both substrates and products than in vacuum, by 17.57 ± 3.26 and 26.14 ± 10.11% for the rTyr and P406L mutant respectively (Figure 2 and Figure S1). The remaining variations between docking in these two environments are depicted in Figure S2. However, these differences may not be significant from the perspective presented in this article; we have opted to showcase only the simulations conducted in a water environment, as this offers a more biologically relevant context.

For rTyr and all of tyrosinase’s substrates, we found a roughly similar number of total ligands docked to the rTyr molecule, from which 2–8% were docked at a distance from copper ions of <4 Å (Figure 2A,B). For L-tyrosine, L-DOPA, DHICA, and DHI, respectively, one, six, five, and two poses were properly oriented, and then, one, one, two, and two stayed in place when the MD simulation was run for 20 ns. It is interesting that, for the products of the rTyr-catalyzed reactions, we found four, one, and two poses for DQ, IQCA, and IQ, respectively, which were <4 Å away from the copper ions; however, none of them were properly oriented toward the active site (Figure 2). Even when the products, such as DQ and IQ, were properly oriented when docking in a vacuum, none remained steady during the MD simulation (Figure S1).

For the substrates of the P406L-catalyzed reactions, similarly to rTyr docking, we found a comparable number of total complex formations; however, only ~1–3% were <4 Å away from the copper ions (Figure 2A,B). Surprisingly, from this pool, only one pose for DHI was properly oriented and simultaneously settled during the MD simulation (Figure 2C,D and Figure S3). Also, for the products of the P406L-catalyzed reactions, two poses for DQ and one pose for IQCA and IQ were found <4 Å away from copper ions. One pose of DQ and IQ was properly oriented toward the active site, however, no steady conformation was found after the MD simulation (Figure S4).

#### 2.2.2. Tyrosinase Substrate Docking Analysis

**Figure 3:**
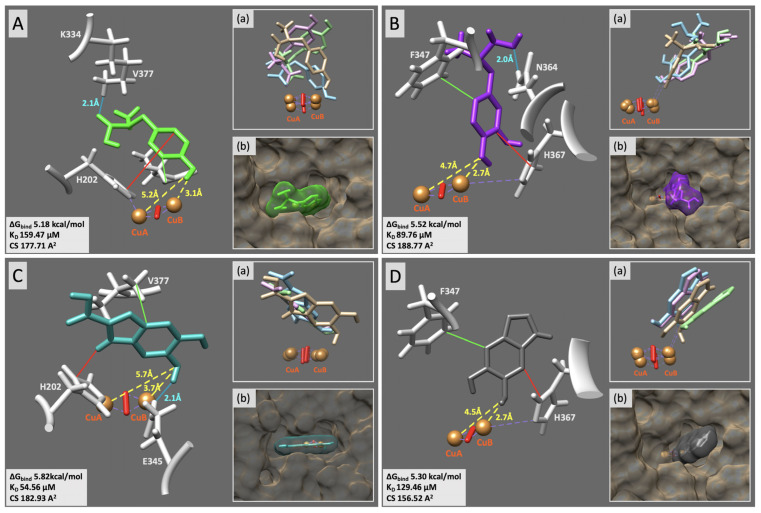
Computational docking of the small-molecule substrates to rTyr. L-tyrosine (green sticks (**A**)), L-DOPA (purple sticks (**B**)), DHICA (green sea sticks (**C**)), and DHI (gray sticks (**D**)) were docked to the rTyr active site. Hydrogen bonds, hydrophobic contacts, and π–π interactions are shown as blue, green, and red lines, respectively. Other contacts for all molecules are shown as purple dashed lines. Residues making any interactions with the docking molecule are shown as light gray sticks. The distances between the docked molecule and the Cu atoms, shown as orange spheres, are colored yellow. Gray boxes show the values of binding energy (ΔG_bind_), dissociation constant (K_D_), and molecular contact surface (CS) between the ligand and receptor. Inserts (**a**) show how the ligand settled during the MD simulation at 0 ns (beige), 1 ns (blue), 10 ns (pink), and 20 ns (green). Inserts (**b**) show the docking of ligands with the surface.

The representative docking of each substrate to rTyr and the interactions between rTyr and the ligands after 1, 10, and 20 ns MD simulations are presented in Figure 3 and Table 1. Additionally, the MD simulation of these substrates was extended to 100 ns to confirm the durability of their complex with rTyr (Figure S5).

For L-tyrosine, L-DOPA, DHICA, and DHI, respectively, the binding energy (ΔG_bind_) values were found to be 5.18, 5.52, 5.82, and 5.30 kcal/mol, and the dissociation constant (K_D_) values were 159, 90, 55, and 129 µM. The molecular contact surface (CS) for the substrates changed from 157 to 189 Å. Also, the phenolic oxygen of rTyr’s substrates was coordinated with the CuB ion, facilitating the hydroxylation or oxidation of the enzymatic reactions. L-tyrosine, in complexes with rTyr, was supported by the hydrogen bonds (HBs) formed between the hydrogen at K334 (donor) and oxygen derived from the L-tyrosine carboxyl group (acceptor), as well as hydrophobic contacts (HpC) with V377 and π–π interactions with H202. However, this complex settled during the MD simulation with L-tyrosine reoriented, displacing oxygen from the aromatic ring from CuB while bringing oxygen from the carboxyl group closer to CuA (Figure 3A, Table 1). L-DOPA was supported by HB formed between the hydrogen at N364 (donor) and oxygen derived from the L-DOPA carboxyl group (acceptor), as well as HpC with F347 and π–π interactions with H367 (Figure 3B). When settled during the MD simulation, the complex was supported by HB formed between the L-DOPA hydroxyl group and Q376 and HpC/π–π interactions with H367 (Table 1). The rTyr/DHICA complex was supported by HB formed between the hydrogen from DHICA’s hydroxyl group (donor) and oxygen at E345 (acceptor), as well as the HpC with V377 and a π–π interaction with H202 (Figure 3C). When settled after MD simulation, the complex was supported by an HB between DHICA and K334, an HpC with V377, and a π–π interaction with H202 (Table 1). The rTyr/DHI complex was supported by an HpC with F347 and a π–π interaction with H367 (Figure 3D). When settled after MD simulation, the complex was supported by an HB between DHI and S375 and a π-π interaction with H367 (Table 1). One more rTyr/DHICA docking pose and one more rTyr/DHI docking pose settled during the MD simulation are presented in Figures S6 and S7, respectively. rTyr/substrate docking poses that properly oriented but did not settle in 20 ns of the MD simulation are shown in Figures S8 and S9.

**Figure 4:**
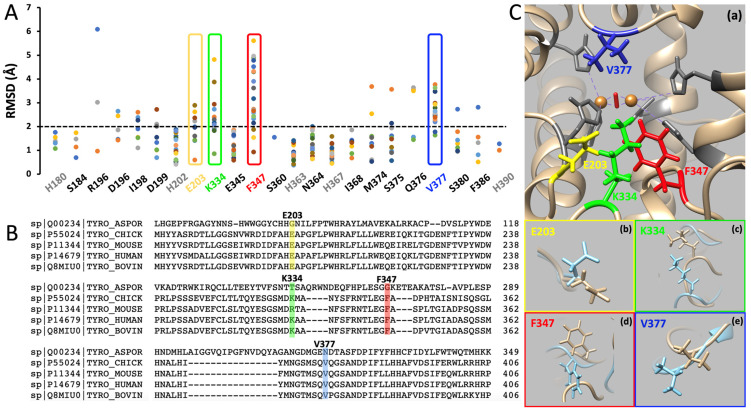
Flexibility of the rTyr/substrates contacting residues after MD simulations. (**A**) RMSD (Å) calculated for the residues involved in docking of all substrates to rTyr after 20 ns of MD simulations. Each dot represents a separate docking action. Histidine-coordinating coppers in the active site are shown in gray. RMSD values < 2 Å (below the black dashed line) are considered stable. The most flexible residues are in the yellow (E203), green (K334), red (F347), and blue (V377) frames. (**B**) Alignment of tyrosinase proteins from fungus, chicken, mouse, human, and bovine. The most flexible residues after 20 ns of MD simulations are shown as yellow (E203), green (K334), red (F347), and blue (V377) bars. (**C**) The homology model of the rTyr domain shows the most flexible residues of E203 (yellow), K334 (green), F347 (red), and V377 (blue) (**a**). Two copper atoms in the active site, CuA and CuB, which are coordinated by histidines (gray), are shown in orange. The protein backbone structure is shown as a tan ribbon. Panels (**b**–**e**) show the movement of E203 (**b**), K334 (**c**), F347 (**d**), and V377 (**e**) residues after 20 ns of MD simulations. Residues in tan indicate the intact rTyr, and blue indicates the supposition of rTyr obtained after docking and 20 ns MD run.

After 20 ns of MD simulations, the root mean square deviation (RMSD) values of the molecules and residues were calculated for the structures in the presence and absence of ligands. The RMSD values calculated for the entire molecule after the docking of L-tyrosine, L-DOPA, DHICA, and DHI were 2.40, 2.61, 2.96, and 2.18 Å, respectively. The RMSD values estimated for each residue involved in the rTyr/substrate docking are shown in Figure 4A. The residues selected by the RMSD criteria in an active site showed significant flexibility in our docking simulations. These flexible residues are conserved in vertebrates, as demonstrated in a sequence alignment (Figure 4B). The location of the flexible residues in an active site of tyrosinase is shown in Figure 4C. The conserved character of the flexible residues indicates the key role of these residues in the binding of multiple substrates.

## 3. Discussion

Small-molecule docking to tyrosinase and MD simulations are computational approaches used in drug discovery and design, particularly in the development of tyrosinase inhibitors, that have potential therapeutic applications in treating hyperpigmentation disorders and skin lightening [12,24,25]. Computational docking using various algorithms and scoring functions is employed to estimate binding affinity and to rank potential inhibitors based on their predicted binding free energy. MD simulations, on the other hand, are used to study the dynamic behavior and conformational changes of protein–ligand complexes at the atomic level. Combining small molecule docking with MD simulations can provide a comprehensive understanding of the binding mechanisms, conformational changes, and thermodynamics of protein–ligand interactions. Here, we use computational methods to study the interactions and dynamics involved in the tyrosinase-catalyzed enzymatic processes of melanin biosynthesis, as well as to understand how missense mutations can affect enzyme–substrate interactions.

It is known that tyrosinase is an enzyme with multiple substrate activities [2,26,27]. Our experiments with rTyr confirmed these activities in vitro. Indeed, the binding of tyrosinase’s substrates, L-tyrosine, L-DOPA, DHICA, and DHI, is specific and involves interactions with key amino acid residues and the copper ions present in the enzyme’s active site. The enzyme–substrate complex does undergo dynamic changes during the catalytic process. We have shown that most of the substrates in the complex with rTyr are stabilized by HB, as well as HpC and π-π interactions with surrounding residues, and some of them stay in the same position during MD simulations (Figure 3, Table 1). A steady rTyr–substrate complex allows the enzyme to properly position the substrate for catalysis and provides a favorable environment for catalytic oxidase reactions to proceed. However, the steadiness of a ligand–enzyme complex after MD simulations is not always necessary. Enzymes may have transient interactions with certain ligands, such as intermediate species in a reaction or allosteric modulators. These interactions can be dynamic and may not result in a steady complex after MD simulations. Tyrosinase catalyzes the transition state of the hydroxylation of L-tyrosine into L-DOPA and the oxidation of L-DOPA into DQ, DHICA into IQCA, and DHI into IQ, facilitating the conversion of the substrate into the product. Once the product is formed, the enzyme may undergo conformational changes to release the product and reset the active site for another catalytic cycle. These conformational changes can cause some docking ligands to shift position during MD simulations. One interesting example is L-tyrosine, which, during MD simulations, turns around, displacing oxygen from the aromatic ring of CuB while bringing oxygen from the carboxyl group closer to CuA (Figure 3A, Table 1). Potentially, it could be related to the fact that tyrosinase shows a lag period in its action on monophenols [28].

rTyr and tyrosinase’s substrates and products may exhibit different levels of conformational flexibility, and rTyr–product complexes may not be as stable as rTyr–substrate complexes. As for the substrates, for the poses < 4 Å away from copper ions, 14–100% of them were properly oriented; for products, it was zero in a water environment and 20–40% in a vacuum (Figure 2 and Figure S1). In addition, 17–100% of properly oriented rTyr–substrate complexes settled during MD simulations, whereas, for rTyr–products complexes, this percentage was zero. rTyr’s active site may adapt its conformation to optimally interact with substrates, leading to steadier complexes. In contrast, the enzyme may not form such optimal interactions with the products, leading to increased conformational flexibility and less firm binding. Similarly, the flexibility of the ligands themselves may differ, with the substrate adopting a more durable conformation in the active site compared with the product.

In P406L, the OCA1B-related mutation in tyrosinase significantly impacts small-molecule substrate binding. We previously showed that the association of L-DOPA with R422Q and P406L mutant variants followed by dopachrome formation is a complex reaction supported by enthalpic and entropic forces [17,18]. rTyr has a higher turnover number as compared with both R422Q and P406L. Although we saw a change in rTyr activity in R422Q and P406L mutant variants, we found similar Km values and thermodynamic behavior for other proteins. This led us to deduce that the change in activity might be due to the allosteric effect [18,29]. Utilizing molecular docking and MD simulations, we demonstrated that, in a water environment, only DHI docked with proper orientation to P406L’s active site and then remained stable after MD simulations (Figure S3). As we measured the enzyme activities for rTyr and P406L in vitro, P406L alteration reduced rTyr activity by 72, 68, 67, and 50% for reactions involving L-tyrosine, L-DOPA, DHICA, and DHI, respectively (Table S2). Residue 406 is located at a considerable distance from the enzyme’s active site and is unlikely to directly alter the shape or orientation of amino acid residues involved in substrate interactions. However, proline is unique among amino acids because its side chain forms a cyclic structure that connects back to the backbone nitrogen. This property restricts the conformational flexibility of the polypeptide chain and often introduces twists or turns in the protein structure. On the other hand, leucine is a nonpolar, hydrophobic amino acid with an aliphatic side chain. The substitution of proline with leucine can cause a change in the local conformation of the protein, which may ultimately affect the overall protein structure, stability, and function. Nevertheless, this mutation can trigger diverse changes. Indeed, according to ClinVar data, P406L is a missense variant with a conflicting classification of pathogenicity.

One of the important questions regarding the melanin pathway is how all tyrosinase substrates bind to it and consequently lead to the formation of different types of melanin. Unfortunately, in the case of rTyr, the conclusion of how multiple substrates interact with tyrosinase is difficult to confirm using biochemical methods. The isothermal titration calorimetry rate for L-DOPA injections in the presence of rTyr is linked to absorbance values ascertained from a Michaelis–Menten plot [17]. This suggests that dopachrome production correlates linearly with the substrate concentration. However, the rTyr-catalyzed conversion of L-tyrosine into dopachrome proceeds through a few steps involving conversions into L-DOPA and dopaquinone, which are difficult to describe in a model of a single binding site. Potentially, in the future, this question could be answered properly in crystallographic experiments on tyrosinase crystals prepared in the presence of its different substrates, but so far, any crystallographic analysis of human tyrosinase has not yet been achieved [30].

If we analyze the details of docking and MD simulations, it becomes apparent that many aspects are shared across all substrates. The π–π interactions between rTyr and its substrates exclusively involve H202 or H367, residues that coordinate CuA and CuB, respectively. Also, the RMSD values calculated for entire molecules before and after docking rTyr’s substrates (2.18–2.96 Å) indicate that the structural changes upon binding are consistent for all substrates. Moreover, the RMSD calculated for all contacting residues between rTyr and its substrates reveals that E203, K334, F347, and V377, the conserved residues in vertebrates, are the most flexible residues during the docking process (Figure 4). This means that these residues can undergo conformational changes or shifts in their spatial arrangements as they interact with ligands and this, in turn, could be a key to understanding how tyrosinase adapts its structure to accommodate and bind different substrates. In prospective studies, experimental design using the site-directed mutagenesis of flexible residues could help to verify, in vitro, whether they indeed play a decisive role in the docking process of all substrates to tyrosinase.

In summary, our study demonstrated that rTyr forms steady complexes with various substrates, crucial for the enzyme’s oxidation function. Interactions with reaction products, if present, are transient, facilitating their release and active site resetting. Furthermore, we highlighted the significance of conserved residues, showing their flexibility during ligand binding, shedding light on tyrosinase’s structural adaptation to different substrates. Our findings suggest that tyrosinase’s flexibility, particularly regarding four conserved residues within its catalytic cavity, may offer opportunities for engineering the enzyme to modulate catalytic activity. The ability of rTyr to selectively bind multiple substrates might reflect the inherent promiscuity of native tyrosinase in melanin biosynthesis. Additionally, we observed that the P406L mutation linked to OCA1B disease selectively affects substrate binding without notable impact on interactions with reaction products, consistent with prior research on the mutation’s effects on tyrosinase activity.

## 4. Materials and Methods

*In vitro analysis of the enzymatic reactions of rTyr and P406L mutant variant.* L-tyrosine, L-DOPA, and DHI were purchased from Sigma-Aldrich (Saint Louis, MO, USA). DHICA was obtained from ChemCruz (Dallas, TX, USA). Recombinant human rTyr (residues 19–469 of the native protein) and P406L mutant variant were expressed in baculovirus and produced in whole-insect *T. ni* larvae (Allotropic Tech, LLC, Halethorpe, MD, USA) and then purified using methods previously described [5,8,31]. rTyr, at a final concentration of 1 mg/mL, and its substrates, L-tyrosine, L-DOPA, DHICA, and DHI, at the concentration of 3, 1.5, 2, and 1.5 mM, respectively, were incubated at 37 °C in 10 mM of NaPO_4_ at pH 7.4 to measure rTyr oxidase activity in absorbance units. The spectrum of absorbance from 200 to 900 nm was recorded using the NanoPhotometer N60-Touch (Implen, Westlake Village, CA, USA). rTyr and P406L mutant variant incubated with L-tyrosine and L-DOPA reached the maximum dopachrome formation (λ_max_ 475 nm) at 180 and 30 min of incubation, respectively. For DHICA, the IQCA formation with λ_max_ 560 nm was measured after 24 h of incubation with rTyr or P406L. For DHI, IQ formation with λ_max_ 327 nm was measured after 60 min of incubation with rTyr or P406L. The significant differences in reaction rates present highly challenging implications for molecular dynamics in the analysis of reasonable chemical reaction timing. Therefore, we opted to use a simplified method to analyze our docking results.

*Molecular Modeling.* A homology model of rTyr (residues 19–469) was built in YASARA [32,33,34] (http://www.yasara.org/, accessed on 1 February 2024) using a crystal structure of Tyrp1 as the structural template (PDB ID: 5M8L) and then glycosylated using the online web tool Glycam-Web (http://glycam.org, accessed on 1 March 2020), as previously described [29]. The P406L mutant variant was generated using the Edit > Swap > Residue function on the 5M8L PDB file in YASARA. The structures were energy-minimized in a water box using the AMBER14 force field [35] and subjected to 100 ns of MD using YASARA’s ‘run.mcr’ macro.

*Docking Experiments.* Five timeframes, 0, 25, 50, 75, and 100 ns, were selected from the MD simulation for the rTyr and P406L mutant variant models. The 2D structure of the small-molecule ligands (L-tyrosine, L-DOPA, DHI, DHICA, DQ, IQ, and IQCA) was retrieved in SDF format from the PubChem compound database (http://pubchem.ncbi.nlm.nih.gov/, accessed on 9 June 2022) or created in Chem Space (https://chem-space.com/search, accessed on 9 June 2022) and then converted to the 3D PDB format in UCSF Chimera [36] (1.16.0, UCSF, San Francisco, CA, USA). Each ligand was docked in two forms, with and without energy minimization, which was performed using AMBER14 with RMSD values of 0.44, 0.23, 0.14, 0.24, 0.17, 0.09, and 0.09 Å for L-tyrosine, L-DOPA, DHICA, DHI, DQ, IQ, and IQCA, respectively. The molecular docking simulations were carried out in both vacuum and water environments (with a water density of 0.997 g/m) using the VINA [37] implemented in YASARA (version 22.9.24, IMBM, University of Graz, Graz, Austria). To set a target, receptors and ligand files were used, and the macro file ‘dock_run.mcr’ was executed. A total of 25 VINA docking runs of the ligand to the receptor were sorted by binding energy (kcal/mol) and dissociation constant (pM) and then clustered, all differing by at least 5.0 Å heavy-atom RMSDs after superposing on the receptor. Point charges and dihedral barriers were obtained from the AMBER03 force field. YASARA was used to analyze all possible interactions between the contacting residues of receptors and ligands, like hydrophobic, Pi-Pi, cation-Pi, and ionic interactions, if the interaction distance was below 5 Å and they were not 1-4 bonded or closer. Hydrogen bonds were identified using the UCSF Chimera software (1.16.0, UCSF, San Francisco, CA, USA) with relaxed hydrogen bond constraints of 0.4 Å and 20 degrees. The protein atomic structures were visualized using UCSF Chimera.

*MD Simulation.* All properly docked structures were subjected to up to 20 ns MD simulations (except for the substrates shown in Figure 3, which were additionally subjected to MD simulations for 100 ns) using YASARA’s ‘runfast’ macro [32]. The simulation temperature was set to 298 K with a water density of 0.997 g/m. The cell size extended to 5 Å beyond each side of the protein in the shape of a cube with dimensions of 102.89 Å × 102.89 Å × 102.89 Å. Each simulation was run in YASARA using an AMBER14 forcefield, with a timestep of 2.5 fs. Simulation snapshots were outputted for every 0.1 ns and then analyzed at 1, 10, and 20 ns for optimal alignment. Additionally, after 20 ns of MD simulations, the structures with and without ligands were superimposed, and the RMSD values of molecules and residues were calculated using the RMSD command in YASARA.

*Analysis of docking and MD simulation results.* In this study, we were interested in discerning differences between substrate binding and product release in tyrosinase enzymatic reactions, as well as differences between docking ligands to the rTyr and P406L mutants. We considered the ligand docked in an active site of rTyr or P406L in the proper orientation when the oxygen from the aromatic ring was oriented toward the binuclear copper location. In addition, the binding of the ligand to the active site was considered valid for protein–ligand interactions if the distance from the oxygen of the hydroxyl or carbonyl group to the CuA or CuB atoms in an active site was below 4.0 Å. All the interactions between rTyr or P406L and their substrates and products were further analyzed using additional MD simulations to investigate the dynamic nature of ligand binding and conformational changes in rTyr/P406L–ligand complexes. We followed the guideline that if the ligand remained in a similar position within 20 ns of an MD simulation without exceeding 4 Å, it was considered an optimal alignment, termed MD settled (steady complex). However, if the ligand does not stay in place in the complex with rTyr or P406L, it is known within the first few femto- or nanoseconds; for several substrates presented in Figure 3, we extended the structural equilibration to 100 ns to confirm the durability of these complexes.

## 5. Conclusions

Our findings contribute to a better understanding of the molecular mechanisms underlying the melanin biosynthesis pathway and have potential implications for the development of therapeutic agents for the OCA type of albinism and other hypopigmentation skin disorders using the intra-melanosomal domain in vitro. Moreover, this study shows the importance of integrating molecular docking and MD simulations in investigating protein–ligand interactions, which could be extended to other biological systems and pathways for further insights into enzyme function and regulation.

## Figures and Tables

**Table 1:** Interactions between rTyr and its docked substrates, L-tyrosine, L-DOPA, DHICA, and DHI after MD simulations at 0, 1, 10, and 20 ns.

Complex	MD (ns)	Interactions			Distance (Å)	
		HB (Distance, Å)	HpC	π–π	CuA	CuB
rTyr/ L-tyrosine	0	K334 1HZ—T O3 (2.1)	T C8—V377 HG1	T C5—H202 CE1	5.2	3.1
	1	S184 HG—T O3 (1.7) T 2HN1—H180 O (2.4) T 3HN1—V377 O (1.8)	T C8—S380 CB	-	4.2	1.6
	10	T 1HN1—D199 OD2 (2.1)	T C7—V377 CG2	-	4.0	2.0
	20	-	T C45—V377 HG1	-	3.9	2.3
rTyr/ L-DOPA	0	N364 HD2—LD O2 (2.0)	LD H5—F347 CE2	LD C6—H367 ND1	4.7	2.7
	1	LD HO1—Q376 O (1.8)	LD C3—H367 CB	LD C9—H367 ND1	4.9	2.7
	10	LD HO1—Q376 O (1.6)	LD C5—H367 CD2	LD C6—H367 ND1	4.9	2.7
	20	LD HO1—Q376 O (1.7)	LD C5—H367 CD2	LD C9—H367 CE1	4.9	2.7
rTyr/ DHICA	0	D H5—E345 OE2 (2.1)	D C2—V377 CG2	D H1—H202 ND1	5.7	3.7
	1	D HO1—V377 O (2.3)	D C5—V377 HG1	D C7—H202 CE1	3.8	2.5
	10	D HO1—V377 O (1.8)	D C6—H202 CD2	D C7—H202 CE1	3.8	2.4
	20	K334 2HZ—D O3 (2.0)	D C5—V377 HG1	D C7—H202 CE1	3.9	2.2
rTyr/ DHI	0	-	DI H3—F347 CE2	DI C8—H367 CD2	4.5	2.7
	1	DI H1—S380 OG (2.4)	DI C4—V377 CG2	DI C3—F347 CE2	4.7	2.7
	10	DI H1—S375 O (1.6)	DI C5—V377 HG2	DI O1—H367 CE1	4.7	2.7
	20	DI H1—S375 O (2.0)	-	DI C8—H367 CD2	4.8	2.7

HB, hydrogen bonds; HpC, hydrophobic contacts; π–π, Pi–Pi interactions; T, L-tyrosine; LD, L-DOPA; D, DHICA; DI, DHI. Distance (Å) was measured between the oxygen of the hydroxyl or carbonyl group of the ligand and the CuA or CuB atoms from the rTyr’s active site.

## Data Availability

The data are available in Supplementary Materials.

## Supplementary Materials

The following supporting information can be downloaded at https://www.mdpi.com/article/10.3390/ijms25063373/s1.

## Abbreviations

OCA: oculocutaneous albinism; OCA1B, oculocutaneous albinism type 1B; P406L, OCA1B-related mutation in *TYR* gene; rTyr, human recombinant intra-melanosomal domain of tyrosinase; L-DOPA, L-3,4-dihydroxyphenylalanine; DQ, dopaquinone; DHICA, 5,6-dihydroxyindole-2-carboxylic acid; IQCA, 5,6-indolequinone-2-carboxylic acid; DHI, 5,6-dihydroxy indole; IQ, indolequinone; MD, molecular dynamics; Tyrp1, tyrosinase-related protein 1; Tyrp2 (DCT), tyrosinase-related protein 2; ΔG_bind_, binding energy, K_D_, dissociation constant; CS, molecular contact surface; HB, hydrogen bond; HpC, hydrophobic contact; π–π, Pi–Pi interactions; RMSD, root mean square deviation.
