# Supplementary material for: Molecular Modeling of the Multiple-Substrate Activity of the Human Recombinant Intra-Melanosomal Domain of Tyrosinase and Its OCA1B-Related Mutant Variant P406L
Source: Int J Mol Sci. 2024 Mar 16;25(6):3373. doi: 10.3390/ijms25063373 (PMC10969854; doi:10.3390/ijms25063373)
Supplement: Supplementary file 1 [file ijms-25-03373-s001.zip › ijms-2833151-supplementary.pdf]

Supporting Material

Table S1. Characterization of the oculocutaneous albinism type 1 – 8.

	OCA									
**Types**	**OCA 1**		**OCA 2**	**OCA 3**		**OCA4**	**OCA5**	**OCA6**	**OCA7**	**OCA8**
**Genes affected**	TYR		OCA2 (P gene)	TYRP 1		SLC45A2 (MATP)	OCA5	SLC24A5 (NCKX5)	C10ORF11	TYRP 2 (DCT)
**Chromosome location**	11q14. 3		15q12-q13.1	9p23		5p13.2	4q24	15q21. 1	10q22.2-q22.3	13q32. 1
**Missense mutations**	419*		265*	67*		149*	5**	21*	30***	3***
**Prevalence**	World: 1/40,000<br>Northern Ireland:<br>1/10,000		World:<br>1/40,000<br>African :<br>1/3,900-1/1,500	World:<br><1 / 1 000 000<br>African:<br>1/8,500		World:<br>1/100,000<br>(more common in Japan)	World:<br><1 / 1 000 000<br>(found only in Pakistani family)	World:<br><1 / 1 000 000<br>(found in 2 Chinese and Indian families)	World:<br><1 / 1 000 000<br>(found in Faroese families (Denmark))	World:<br><1 / 1 000 000<br>(several cases only)
**Inheritance**	Autosomal recessive									
**subtypes**	**OCA 1A**	**OCA 1B**	N/A	**ROCA**	**BOCA**	N/A	N/A	N/A	N/A	N/A
**Pigmentation phenotype**	No pigment	SHEP3:<br>light/dark skin<br>freckling skin<br>blue/green eye	SHEP1:<br>blue/brown eyes<br>blond/brown hair	SHEP11:<br>Melanesian blond hair<br>blue/nonblue eyes		SHEP5:<br>black/nonblack hair<br>dark/light eyes<br>dark/fair skin		SHEP4:<br>fair/dark skin		
**Clinical Features**	•white skin and hair at birth<br>•Irises are blue to pink and fully translucent. These features do not change throughout a patient's life.	•white or very light-yellow hair that darkens with age<br>•Creamy skin but a minimal amount of tanning is possible along with freckles and pigmented nevi<br>•Blue iris can change to brownish tan or greenish hazel or remain unchanged	•variable hypopigmentation of the skin and hair	•red-bronze skin<br>•blue or brown irises<br>•ginger-red hair	•light to brown hair<br>•light to brown or tan skin	•varying degrees of skin and hair hypopigmentation	•white skin<br>•golden hair	•light hair at birth that darkens with age<br>•white skin	•skin and hair hypopigmentation (light blond to dark brown)	•mild hypopigmentation of the skin, hair, and eyes
**Ocular changes**	•Photophobia<br>•Nystagmus<br>•Strabismus<br>•foveal hypoplasia<br>•impaired visual acuity	•misrouting of the optic nerves at the chiasm<br>•Nystagmus<br>•Strabismus<br>•Foveal hypoplasia<br>•reduction in visual acuity<br>•Photophobia	•misrouting of the optic nerves at the chiasm<br>•pink-eyed dilution<br>•transillumination<br>•hypoplastic maculae<br>•Nystagmus<br>•severe myopia<br>•Strabismus	•Photophobia<br>•Strabismus<br>•impaired visual acuity		•misrouting of the optic nerves at the chiasm<br>•Nystagmus<br>•Strabismus<br>•Foveal hypoplasia<br>•reduction in visual acuity<br>•Photophobia	•Photophobia<br>•Nystagmus<br>•foveal hypoplasia<br>•impaired visual acuity	•transparent irides<br>•Photophobia<br>•nystagmus<br>•foveal hypoplasia<br>•reduced visual acuity	•Nystagmus<br>•iris transillumination<br>•reduction in visual acuity<br>•hypopigmentation of the peripheral ocular fundus	•moderate reduction of visual acuity<br>•nystagmus<br>•foveal hypoplasia<br>•iris transillumination<br>•hypopigmentation of the retina

Based on the Orphanet resource (https://www.orpha.net/consor/cgi-bin/index.php) and OMIM catalog of Human Genes and Genetic Disorders (https://omim.org).

*HGMD database (https://www.hgmd.cf.ac.uk/ac/index.php)

** Kausar et al., 2013(1)

***Clinvar database (https://www.ncbi.nlm.nih.gov/clinvar/)

Table S2. The activity of the rTyr and P406L mutant variant was measured as the maximum absorbance of dopachrome, IQCA, and IQ, products of the reactions with L-tyrosine or L-DOPA, DHICA, and DHI, respectively.

Substrates	Absorbance (U)		% of rTyr
	rTyr	P406L	
**L-tyrosine**	3.76 ± 0.06	1.07 ± 0.02	28
**L-DOPA**	1.75 ± 0.18	0.56 ± 0.16	32
**DHICA**	4.70 ± 0.06	1.55 ± 0.05	33
**DHI**	5.51 ± 0.17	2.77 ± 0.16	50

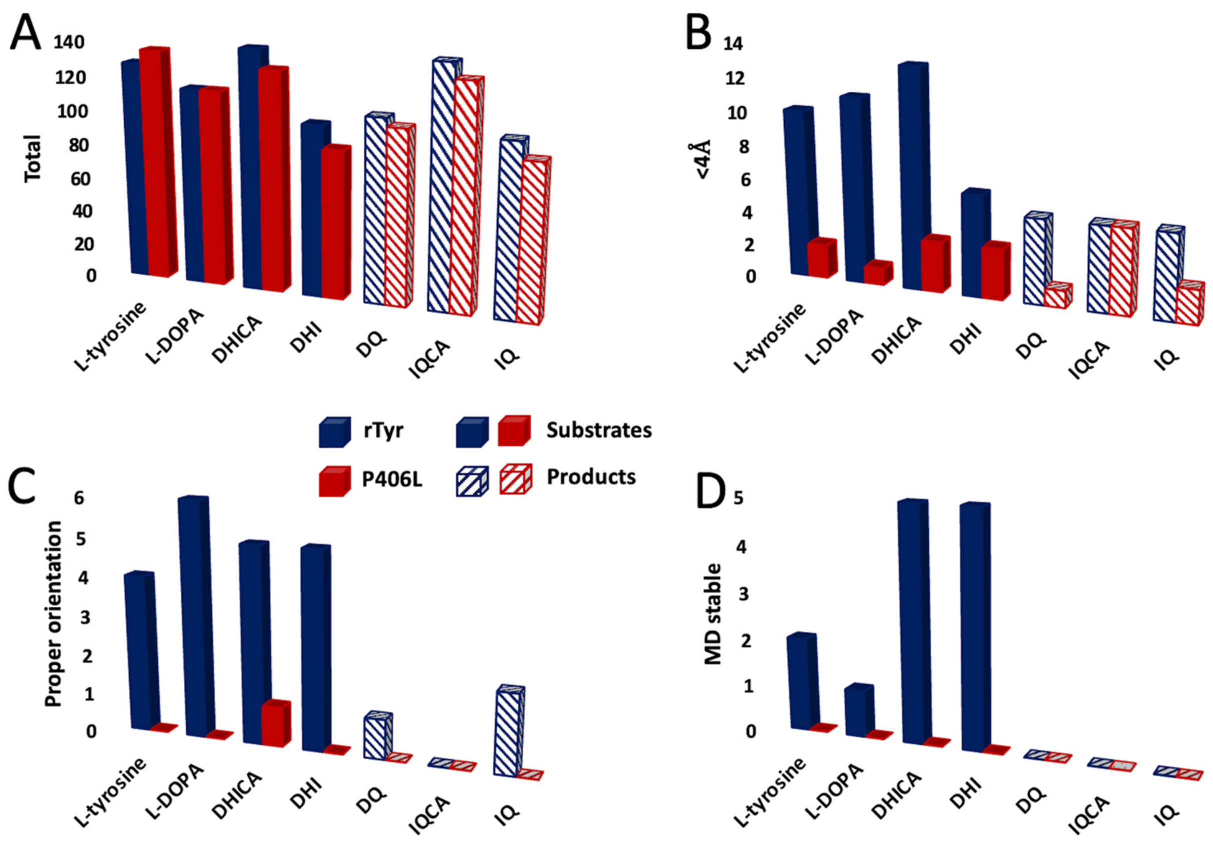

Figure S1. Molecular docking of the substrates and products of the enzymatic reaction catalyzed by rTyr and P406L mutant variant simulated in the vacuum. Panel A: Number of complex formations (total) found after clustering the 25 docking runs of the ligands: substrates (solid bars) and products (stripped bars) to the rTyr (blue) and P406L (red). **Panel B** shows number of the ligands bound to the active site in the distance below 4.0 Å from the CuA or CuB atoms. **Panel C** shows the number of ligands of which the oxygen of the hydroxyl or carbonyl group is directed towards the active site (proper orientation). **Panel D** shows the number of all properly docked structures stable after 20 ns of MD simulations.

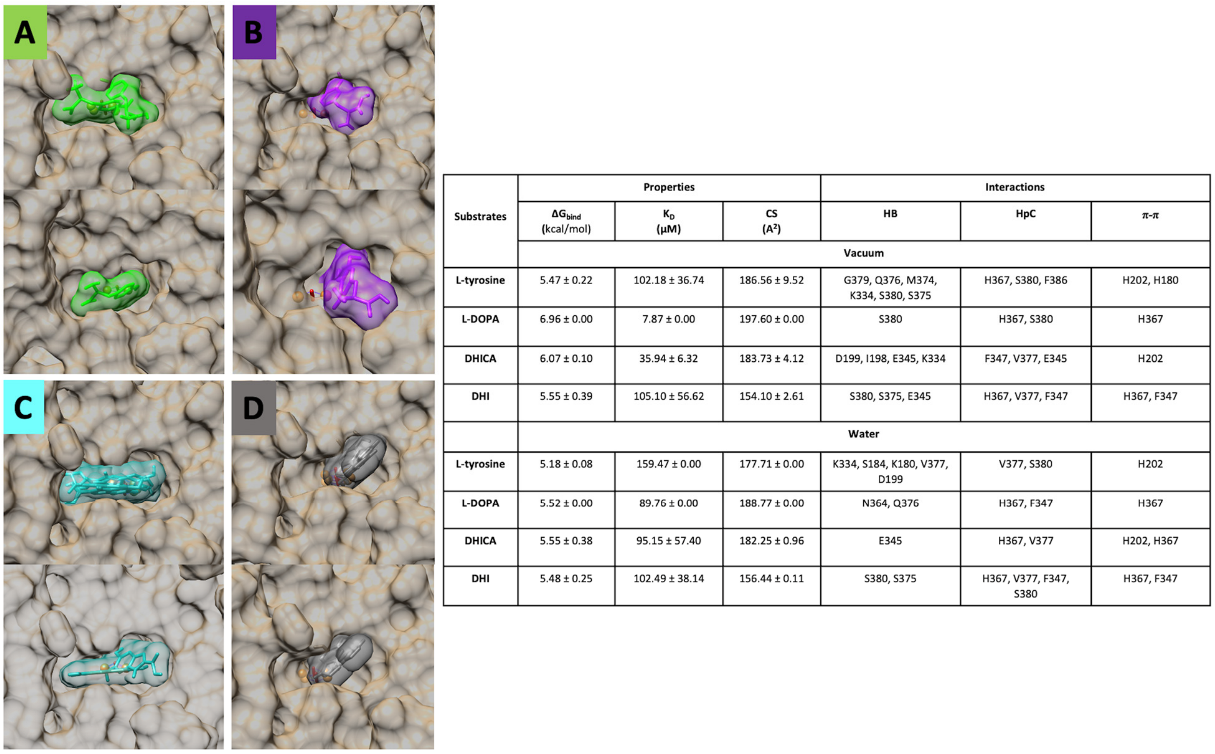

Substrates	Properties			Interactions		
	ΔG_{bind} (kcal/mol)	K_D (μM)	CS (A^2)	HB	HpC	π-π
	Vacuum					
**L-tyrosine**	5.47 ± 0.22	102.18 ± 36.74	186.56 ± 9.52	G379, Q376, M374, K334, S380, S375	H367, S380, F386	H202, H180
**L-DOPA**	6.96 ± 0.00	7.87 ± 0.00	197.60 ± 0.00	S380	H367, S380	H367
**DHICA**	6.07 ± 0.10	35.94 ± 6.32	183.73 ± 4.12	D199, I198, E345, K334	F347, V377, E345	H202
**DHI**	5.55 ± 0.39	105.10 ± 56.62	154.10 ± 2.61	S380, S375, E345	H367, V377, F347	H367, F347
	Water					
**L-tyrosine**	5.18 ± 0.08	159.47 ± 0.00	177.71 ± 0.00	K334, S184, K180, V377, D199	V377, S380	H202
**L-DOPA**	5.52 ± 0.00	89.76 ± 0.00	188.77 ± 0.00	N364, Q376	H367, F347	H367
**DHICA**	5.55 ± 0.38	95.15 ± 57.40	182.25 ± 0.96	E345	H367, V377	H202, H367
**DHI**	5.48 ± 0.25	102.49 ± 38.14	156.44 ± 0.11	S380, S375	H367, V377, F347, S380	H367, F347

Figure S2. The properties of docking of rTyr's substrates in vacuum or water environments. The pictures show all properly oriented docking poses obtained after docking simulation in vacuum (**top panels**) or water (**bottom panels**) environments. The ligands are L-tyrosine (green, **A**), L-DOPA (purple, **B**), DHICA (sea green, **C**), and DHI (grey, **D**). rTyr surface is colored tan. The table shows the properties of binding: binding energy (ΔG_{bind}), dissociation constant (K_D), molecular contact surface (CS), and interactions: hydrogen bonds (HB), hydrophobic contacts (HpC), and Pi-Pi interactions (π-π) between the ligands and receptor.

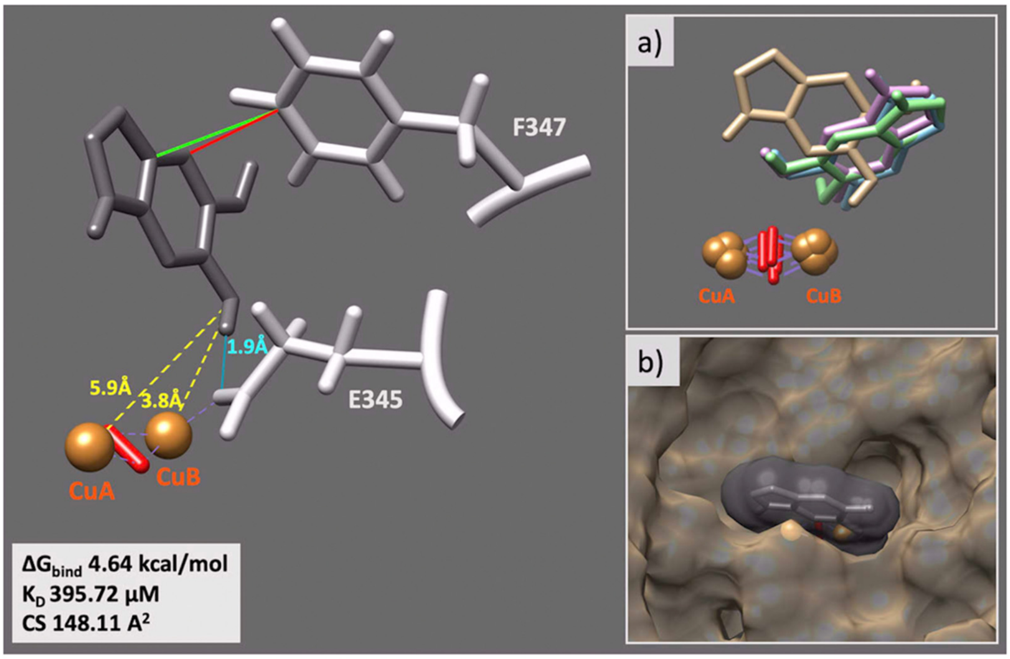

MD (ns)	Interactions			Distance (Å)	
	HB	HpC	π-π	CuA	CuB
0	DI H1 – E345 OE2 (1.9 Å)	DI C4 – F347 CZ	DI C3 – F347 CZ	5.9	3.8
1	-	DI C3– H367 CD2	DI C3– H367 CD2	3.6	3.0
10	-	DI C3– H367 CD2	DI C3– H367 CD2	3.5	2.9
20	-	DI C8 – E345 CG	DI C3– H367 CD2	4.2	1.9

Figure S3. Computational docking of DHI to the P406L mutant variant stable after MD simulation. DHI (grey stick) is stabilized by the hydrogen bond (blue line) with E345 as well as the hydrophobic contact (green line) and π-π interaction (red line) with F347. Other contacts are shown by purple dashed lines. Residues making any interaction with DHI are shown by light grey sticks. The distances between DHI and Cu atoms, shown as orange spheres, are colored yellow. The grey box shows the values of binding energy (ΔG_{bind}), dissociation constant (K_D), and molecular contact surface (CS) between the ligand and receptor. **Insert a)** shows DHI movement during the MD after 0 ns (beige), 1 ns (blue), 10 ns (pink), and 20 ns (green). **Insert b)** shows the docking DHI with the surface (grey). **The table** shows the hydrogen bonds (HB), hydrophobic contacts (HpC), and Pi-Pi interactions (π-π), between P406L and docked DHI (DI) and the corresponding distances between DHI and Cu atoms after MD simulations at 0, 1, 10, and 20 ns.

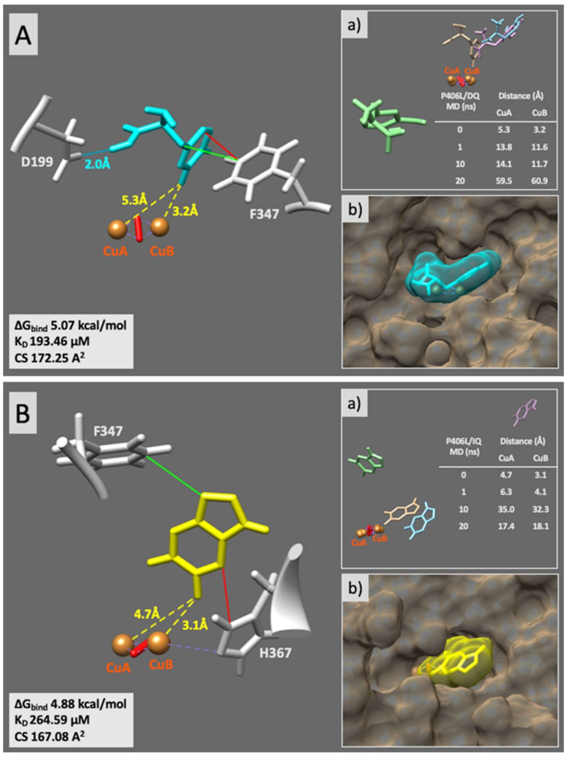

Figure S4. Molecular docking results for the products of reactions catalyzed by the P406L mutant variant. Dopaquinone (DQ, cyan stick) (**Panel A**) and IQ (yellow stick) (**Panel B**) were docked to the P406L active site. Hydrogen bonds, hydrophobic contacts, and π-π interactions are shown as blue, green, and red lines, respectively. Other contacts for all molecules are shown by purple dashed lines. Residues making any interaction with the docking molecule are shown by light grey sticks. The distances between the docked molecule and Cu atoms, shown as orange spheres, are colored yellow. The grey boxes show the values of binding energy (ΔG_{bind}), dissociation constant (K_D), and molecular contact surface (CS) between the ligand and receptor. **Inserts a)** show the small molecules' movement during the MD simulation after 0 ns (beige), 1 ns (blue), 10 ns (pink), and 20 ns (green). Tables show the distances between docking molecules and Cu atoms after MD simulations at 0, 1, 10, and 20 ns. **Inserts b)** show the dopaquinone (cyan) and IQ (yellow) docking molecules with the surface.

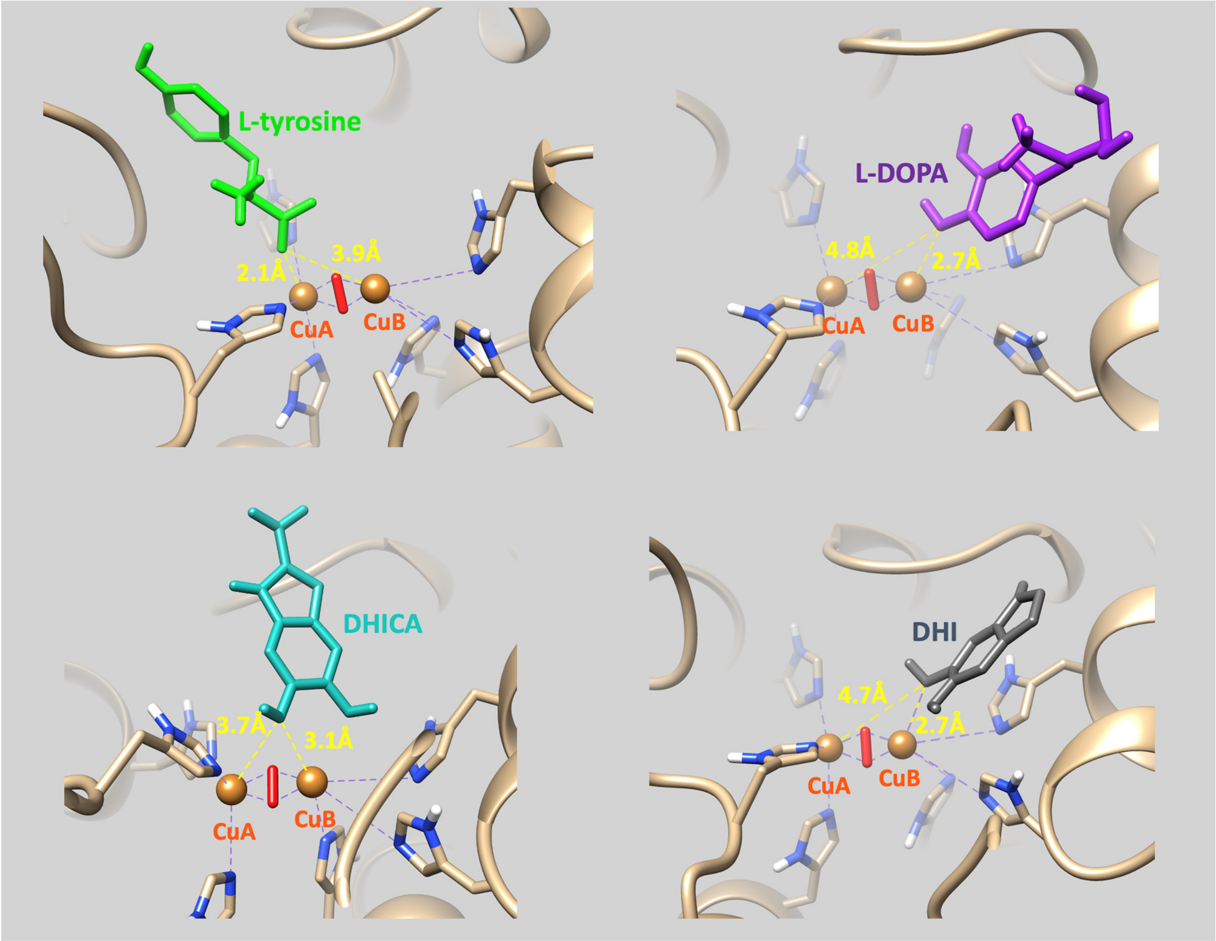

Figure S5. Computational docking of the small molecule substrates to rTyr after 100 ns of MD simulation. L-tyrosine (green stick), L-DOPA (purple stick), DHICA (green sea stick), and DHI (grey stick) were docked to the rTyr active site and undergoes 100 ns of MD simulation. The protein backbone structure is shown by a tan ribbon. Two copper atoms in the active site, CuA and CuB, which were coordinated by histidines are shown by orange spheres. The distances between the docked molecule and Cu atoms are colored yellow. Other contacts for all molecules are shown by purple dashed lines.

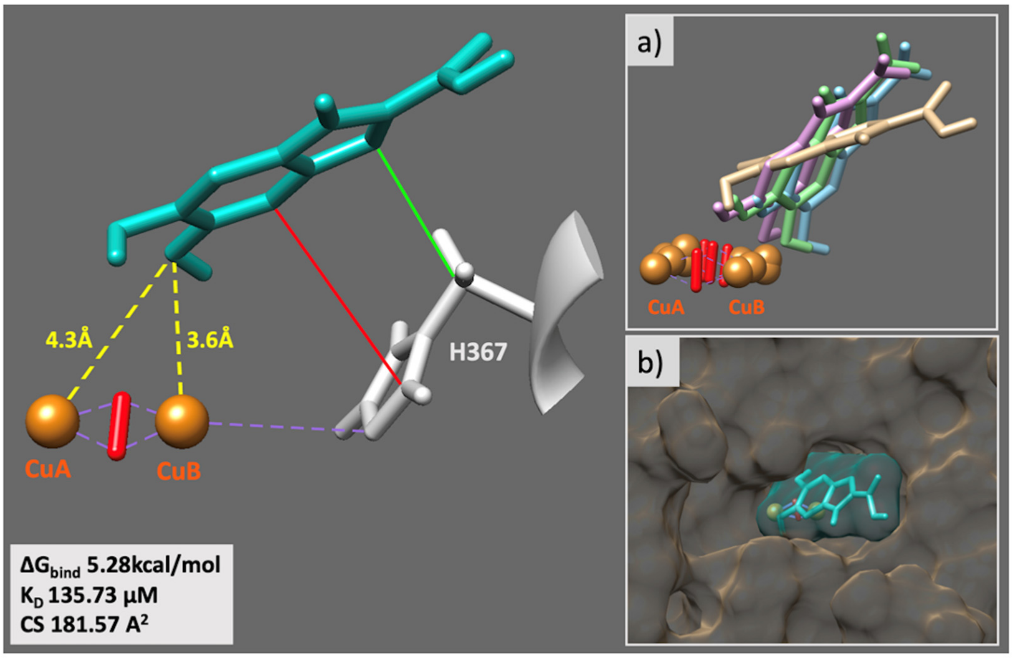

MD (ns)	Interactions			Distance (Å)	
	HB	HpC	π-π	CuA	CuB
0	-	D C3 – H367 CB	D C6 – H367 CD2	4.3	3.6
1	-	D C6 – H367 CD2	D H6 – H367 ND1	4.3	1.7
10	-	D C6 – H367 CD2	D C6 – H367 CE1	4.1	1.6
20	-	D C6 – H367 CD2	D C6 – H367 CE1	4.2	1.6

Figure S6. Computational docking of DHICA to rTyr stable after MD simulation. DHICA (green sea stick) is stabilized by hydrophobic contact (green line) and π-π interaction (red line) with H367 residue (light grey stick). Other contacts are shown by purple dashed lines. The distances between DHICA and Cu atoms, shown as orange spheres, are colored yellow. The grey box shows the values of binding energy (ΔG_{bind}), dissociation constant (K_D), and molecular contact surface (CS) between the ligand and receptor. **Insert a)** shows DHICA stability after the MD simulation at 0 ns (beige), 1 ns (blue), 10 ns (pink), and 20 ns (green). **Insert b)** shows the docking DHICA with the surface (green sea). **The table** shows the hydrogen bonds (HB), hydrophobic contacts (HpC), and Pi-Pi interactions (π-π), between Tyr and docked DHICA (D) and the distances between DHICA and Cu atoms after MD simulations at 0, 1, 10, and 20 ns.

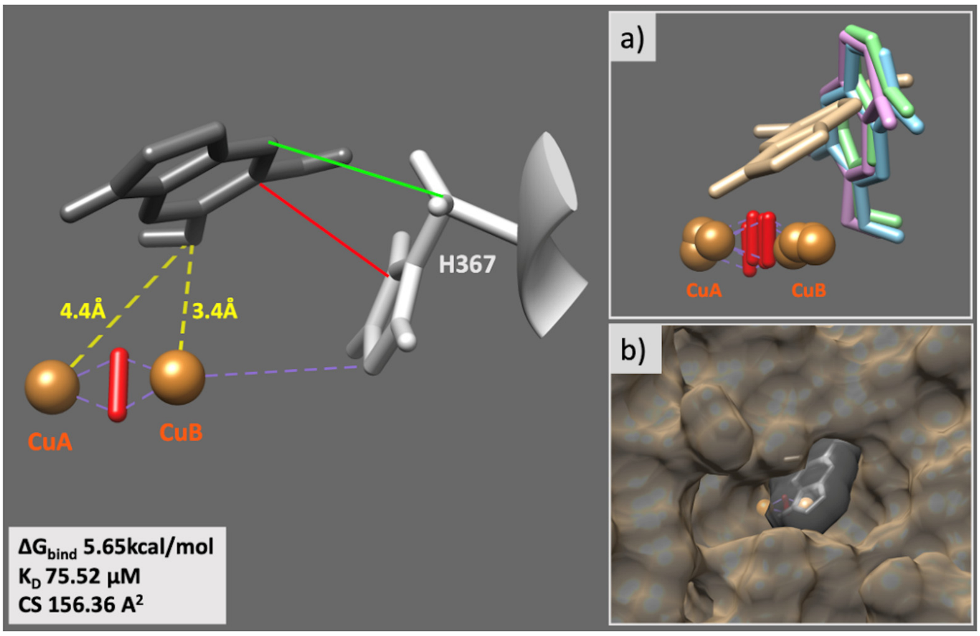

MD (ns)	Interactions			Distance (Å)	
	HB	HpC	π-π	CuA	CuB
**0**	-	DI C3 – H367 CB	DI C2 – H367 ND1	4.4	3.4
**1**	DI H2 – S380 OG (1.8 Å)	DI C3– S380 CB	DI C8 – H367 CE1	4.2	1.6
**10**	DI H2 – S380 OG (2.0 Å)	DI C8 – H367 CB	DI C1 – H367 CE1	4.2	1.5
**20**	DI H2 – S380 OG (1.8 Å)	DI H5 – V377 CG2	DI C1 – H367 CE1	4.3	1.6

Figure S7. Computational docking of DHI to rTyr stable after MD simulation.

DHI (grey stick) is stabilized hydrophobic contact (green line) with an π-π interaction (red line) with H367 (light grey stick). Other contacts are shown by purple dashed lines. The distances between DHI and Cu atoms, shown as orange spheres, are colored yellow. The grey box shows the values of binding energy (ΔG_{bind}), dissociation constant (K_D), and molecular contact surface (CS) between the ligand and receptor. **Insert a)** shows DHI stability after the MD simulation at 0 ns (beige), 1 ns (blue), 10 ns (pink), and 20 ns (green). **Insert b)** shows the docking DHI with the surface (grey).

The table shows the hydrogen bonds (HB), hydrophobic contacts (HpC), and Pi-Pi interactions (π-π), between rTyr and docked DHI (DI) and the distances between DHI and Cu atoms obtained after MD simulations at 0, 1, 10, and 20 ns.

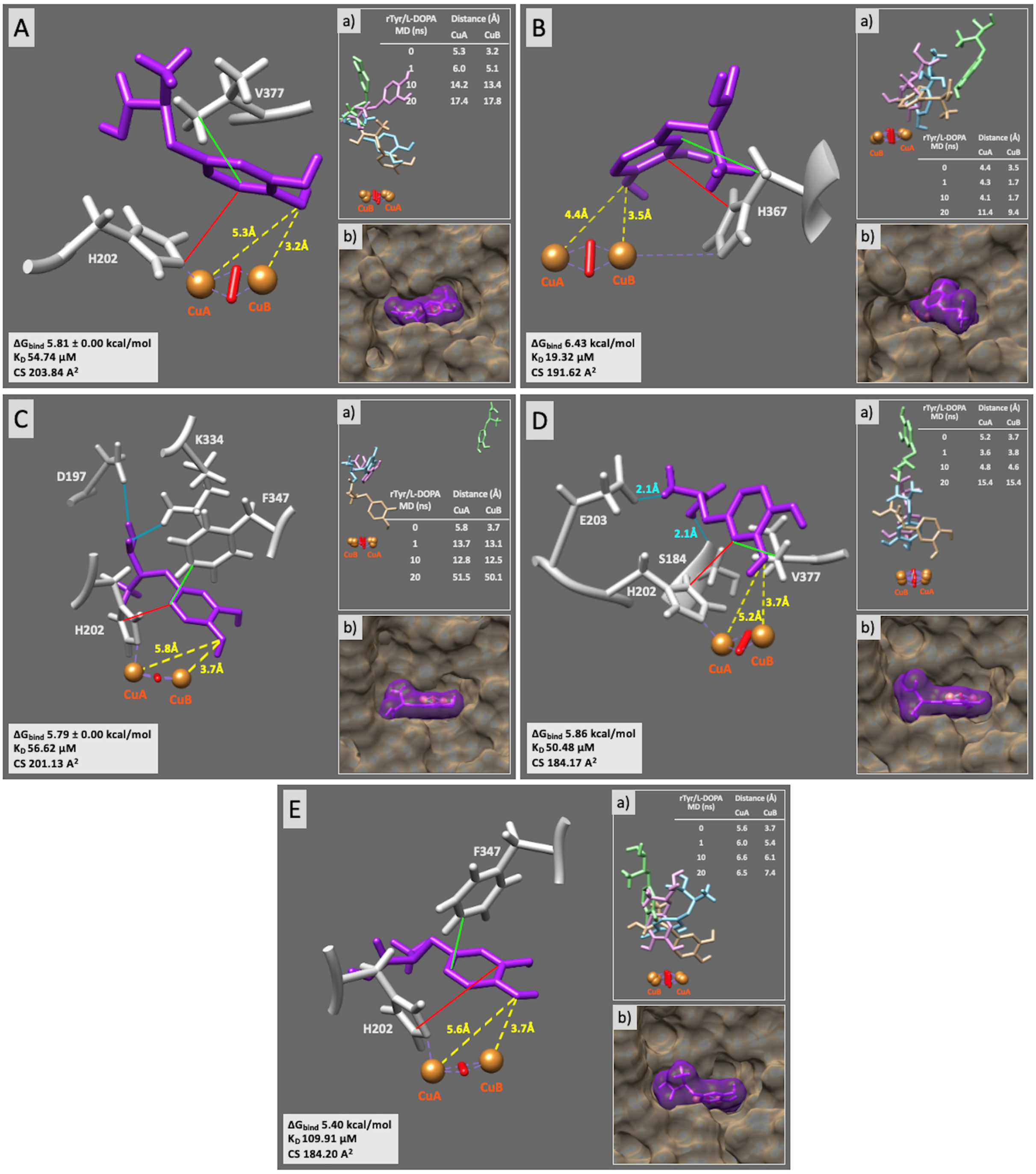

Figure S8. Computational docking of L-DOPA to rTyr unstable after MD simulation. **Panel A:** L-DOPA (purple stick) is stabilized by hydrophobic contact (green line) with V377 and π-π interaction (red line) with H202. **Panel B:** L-DOPA is stabilized by hydrophobic contact and π-π interaction with H367. **Panel C:** L-DOPA is stabilized by two hydrogen bonds (blue lines) with D197 and K334 residues, as well as by hydrophobic contact with F347 and π-π interaction

with H202. **Panel D:** L-DOPA is stabilized by two hydrogen bonds with E203 and S184 residues, as well as by hydrophobic contact with V377 and π-π interaction with H202. **Panel E:** L-DOPA is stabilized by hydrophobic contact with F347 and π-π interaction with H202. Other contacts are shown by purple dashed lines. Residues making any interaction with L-DOPA are shown by light grey sticks. The distances between L-DOPA and Cu atoms, shown as orange spheres, are colored yellow. Grey boxes show the values of binding energy (ΔG_{bind}), dissociation constant (K_D), and molecular contact surface (CS) between the ligand and receptor. **Inserts a)** show L-DOPA movement during the MD after 0 ns (beige), 1 ns (blue), 10 ns (pink), and 20 ns (green). Tables show the distances between L-DOPA and Cu atoms after MD simulations at 0, 1, 10, and 20 ns. **Inserts b)** show the docking L-DOPA (purple) with the surface.

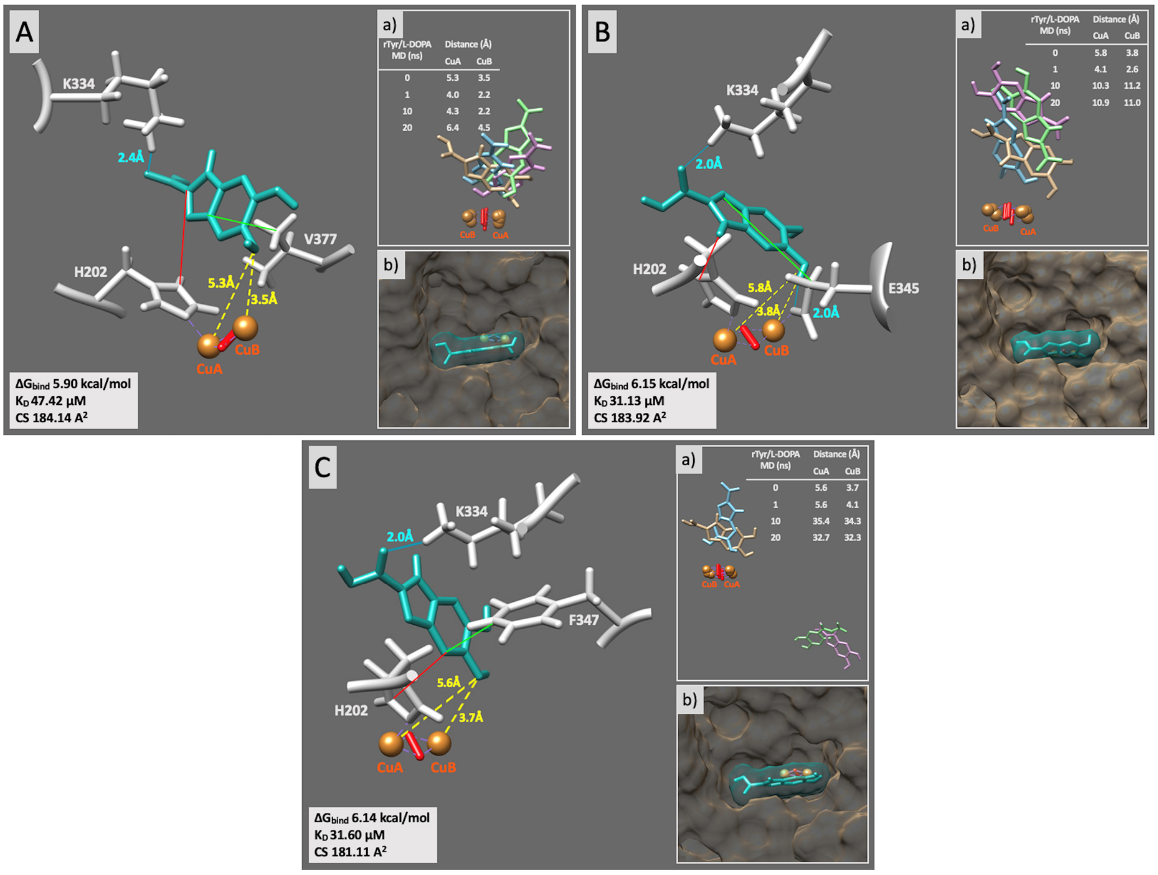

Figure S9. Computational docking of DHICA to rTyr unstable after MD simulation. **Panel A:** DHICA (green sea stick) is stabilized by the hydrogen bond (blue line) with K334, hydrophobic contact (green line) with V377, and π-π interaction (red line) with H202. **Panel B:** DHICA is stabilized by two hydrogen bonds with K334 and E345, as well as the hydrophobic contact with E345 and π-π interaction with H202. **Panel C:** DHICA is stabilized by the hydrogen bond with K334, hydrophobic contact with F347, and π-π interaction with H202. Other contacts are shown by purple dashed lines. Residues making any interaction with DHICA are shown by light grey sticks. The distances between DHICA and Cu atoms, shown as orange spheres, are colored yellow. The grey box shows the values of binding energy (ΔG_{bind}), dissociation constant (K_D), and molecular contact surface (CS) between the ligand and receptor. **Inserts a)** show DHICA movement during the MD simulation after 0 ns (beige), 1 ns (blue), 10 ns (pink), and 20 ns (green). The table shows the distances between DHICA and Cu atoms obtained after MD simulations at 0, 1, 10, and 20 ns. **Inserts b)** show the docking DHICA (green sea) with the surface.

References

1. Kausar T, Bhatti MA, Ali M, Shaikh RS, Ahmed ZM. OCA5, a novel locus for non-syndromic oculocutaneous albinism, maps to chromosome 4q24. Clin Genet. 2013;84(1):91-3.
